# A Household Word

**Published:** 1870-09

**Authors:** 


### A HOUSEHOLD WORD.

A CORRESPONDENT writes, "Our little six year old daughter refused to eat a 'piece' near supper-time, saying, 'Dr. Hall says we should not eat between meals.'"

Many say and write, that the teachings of the *Journal* are considered a law to themselves, and in their households; that "Dr. Hall's sayings are household words." These things do not gladden, but sadden, by the flitting thought of responsibility; and then comes the fear that now and then we may teach some error. And what must it be with the clergyman, whose teachings, humanly speaking, may draw the inquiring to a path, which, diverging from the truth, leads the soul further away forever!

What must it be with the honorable lawyer, where omission of a phrase or word, or where ignorance or mistake as to a point of law, may lose his client large sums of money; we knew a case which cost a client's life.

### THE NOBLE LAWYER.

We read of a case some years ago, so peculiar in its nature, that the omission of the letter *s* in a legal document, in the English courts, cost the client thirty thousand pounds, or one hundred and fifty thousand dollars; it was brought to the lawyer's attention; there could be no mistake; in a moment he sat down and wrote a check for the amount, in his client's favor.

We take occasion here to add to the impressiveness of our health teachings, by the statement of the fact, that they are not ours, in the sense either of research, discovery, or induction; we have only aimed to put in familiar words, the truths, and facts, and natural laws, brought to light by the hard studies, the laborious investigations and destructive labors of the greatest medical minds of the past ages, and up to the present time. We say "destructive labor," for quite a number of the great men devoted to medical science have died prematurely, by accidents in the chemical laboratory, in the examination of poisonous dead bodies, and in experiments on untried remedies. But men of great minds like these, have not the time, nor the taste, nor indeed the ability to descend to the drudgery of explaining first principles, and of manufacturing brains for people who haven't got any; or if they have, can't use them to any beneficially practical extent, because they won't take time to think or see. So we have taken it upon ourself, in writing for the *Journal of Health*, simply to select some of the most important ascertained truths belonging to human health, and put them in language so plain, that it does not give one the headache in studying out what it means. All that is required of any writer to be successful, to be popular, is to be able to catch the reader's apprehension as he runs, without the effort of thinking; but mind, the thing must be solid, sensible, and useful.

